# Ezetimibe Anticancer Activity via the p53/Mdm2 Pathway

**DOI:** 10.3390/biomedicines13010195

**Published:** 2025-01-14

**Authors:** Charmy Twala, Sibusiso Malindisa, Chamone Munnik, Selisha Sooklal, Monde Ntwasa

**Affiliations:** 1 Department of Life & Consumer Sciences, College of Agriculture and Environmental Sciences, University of South Africa, Cnr. Pioneer and Christiaan de Wet Roads, B2-010 Calabash Building, Florida, Johannesburg 1710, South Africa; twalacs@unisa.ac.za (C.T.); malinst@unisa.ac.za (S.M.); 63280086@mylife.unisa.ac.za (C.M.); sooklsa@unisa.ac.za (S.S.); 2 Buboo Bioinnovations (Pty) Ltd., The Innovation Hub, Hatfield, Pretoria 0200, South Africa

**Keywords:** p53, Mdm2, drug design, drug development, drug repurposing, Ezetimibe, cancer

## Abstract

Background: Ezetimibe is used to treat cardiovascular disease as it blocks the sterol transporter Niemann-Pick C1-Like 1 (NPC1CL1) protein. However, recent evidence indicates that Ezetimibe inhibits several cancers indirectly by reducing circulating cholesterol or via specific signalling pathways. Methods and Results: Our in silico studies indicate that Ezetimibe binds to the Tp53 binding domain in Mdm2, forming a more thermodynamically stable complex than nutlin3a. Furthermore, a docking study of the newly developed inhibitors—RG7388 and RG7112—was conducted. This further showed lower binding energies of −6.337 kcal/mol and −6.222 kcal/mol, respectively, when compared to the −7.919 kcal/mol exhibited by Ezetimibe. We show that Ezetimibe inhibits the growth of several cancer cell lines at concentrations that are not toxic to a normal cell line. Conclusions: Thus, Ezetimibe is probably active against cancers that overexpress Mdm2. Moreover, inhibitors of RBBP6 may be combined with Ezetimibe for effective anticancer activity. Due to poor oral bioavailability, Ezetimibe must be administered parenterally for cancer treatment.

## 1. Introduction

Ezetimibe is a United States Food and Drug Administration (FDA)-approved drug that blocks cholesterol uptake in the intestines, thereby reducing its concentration in the systemic circulation. It is thus used to treat hypercholesterolemia. Orally administered Ezetimibe is rapidly glucuronidated and recycled by the enterohepatic circulation to its target. It is reported that this glucuronide metabolite is at least as potent as the parent drug in inhibiting cholesterol uptake. This is based on experiments using the initial drug lead, SCH48461, which inhibited cholesterol absorption by 70%, whereas the metabolite inhibited absorption by more than 95%. Furthermore, the metabolite was retained on the intestinal wall, and the bulk of it was not systemically available.

The known target of Ezetimibe is the *Niemann–Pick C1-Like 1* (NPC1L1) found in jejunal enterocytes. Up to 80% of Ezetimibe is rapidly metabolized to its pharmacologically active glucuronide metabolite (EZE-GLUC) by uridine 5-diphosphate (UDP)-glucuronosyl-transferase (UGT) 1A1, 1A3, and 2B15 in the intestine. The remaining parent drug and the glucuronidated metabolite are then excreted into the bile via the portal vein and delivered back into the intestinal site of action, thereby increasing the drug’s half-life. Cholesterol absorption studies indicated that the glucuronide appeared more potent than Ezetimibe itself because glucuronidated Ezetimibe localizes more avidly to the intestine. Ezetimibe and/or the glucuronide metabolite are excreted in feces (90%) and urine (10%).

The Niemann-Pick C1-Like 1 receptor was identified as a target for Ezetimibe through a systematic search of transcriptomics databases. This discovery was supported by showing that NPC1CL1^−/−^ mice were substantially defective in cholesterol absorption and were resistant to Ezetimibe [1]. Subsequently, a binding assay showed the direct association between Ezetimibe and NPC1CL, by confirming that glucuronidated Ezetimibe binds to the receptor [2].

Experimental evidence has recently supported Ezetimibe as a potential anticancer drug [3]. Some evidence is indirect and based on the function of NPC1L1 as a receptor that mediates cholesterol uptake in the small intestine. For example, NPC1L1^−/−^ mice were found to develop fewer tumours when compared with wild-type mice after the induction of colitis-associated colorectal tumours [4]. In Pancreatic Ductal Adenocarcinoma expressing NPC1CL1, Ezetimibe was toxic and had no effect on fibroblasts [5]. Furthermore, PDAC cells where NPC1L1 was knocked down by siRNA, were resistant to high Ezetimibe concentrations. In another study, Ezetimibe inhibited prostate tumour growth by reducing serum cholesterol, thereby inhibiting angiogenesis [6]. Recent studies show that Ezetimibe promotes mitochondrial dysfunction, cell death, and cell cyle arrest. In a colorectal cancer cell line, Ezetimibe induced autophage-associated apoptosis and was associated with the mTOR signalling pathway [7]. In another study, Ezetimibe was proposed as a possible treatment for triple negative breast cancer as it blocked the cell cycle in the G1 phase and actvated the PDGFRβ/AKT pathway [8].

The tumour protein (p53) affects several necessary biological processes that are crucial for carcinogenesis, including the cell cycle, apoptosis, DNA repair, angiogenesis, glucose metabolism, and innate immunity [9,10]. A previous study showed that Ezetimibe binds snugly into the p53-binding domain of the Mouse Double Minute (Mdm2) protein [11]. p53 is a transcription factor that functions in an autoregulatory feedback loop with ubiquitin-ligase activity. Thus, as a transcription factor, p53 activates the expression of the gene. On the other hand, Mdm2 regulates p53 by controlling its transport out of the nucleus, making it unavailable to gene targets, inhibiting its transcription function, or promoting its degradation by the proteasome using its Ubiquitin-ligase activity. It has been shown in previous studies that the Retinoblastoma Binding Protein 6 (RBBP6) has a similar phenotype to Mdm2 when deleted in mice. Both *mdm2^−/−^* and *rbbp6*^−/−^ embryos die, a phenotype that a deletion of the p53 gene can rescue. RBBP6 is the p53-associated cellular protein of testes derived (PACT) in mice. However, the *rbbp6*^−/−^ phenotype is more severe when compared to the *mdm2*^−/−^, indicating that RBBP6 also functions in pathways independent of Mdm2 [12]. Moreover, RBBP6 is reported to be a prognostic marker and an inducer of cancers such as colorectal and non-small lung cancer [13,14]. Taken together, these findings constitute strong evidence about the druggability of the p53/RBBP6/Mdm2 complex.

Mdm2 has a hydrophobic binding pocket, to which p53 binds through a peptide in its transactivation domain [15]. This pocket is, therefore, a key target for drugs that inhibit the p53-Mdm2 interaction. Indeed, several small molecular drug design studies formulated molecules that can competitively target the Mdm2 p53-binding domain disrupting the formation of Mdm2-p53 complexes and, thus, reactivate p53 levels in cancer cells to promote p53-dependent cell death [16]. In the current study, we provide data reinforcing that RBBP6 may be a scaffold protein that forms a complex with Mdm2 and p53. This suggests that combined targeting of Mdm2 and RBBP6 would produce a good treatment for cancers that overexpress these molecules.

## 2. Materials and Methods

### 2.1. Drugs

Ezetimibe (SML1629 Sigma-Aldrich, Saint Louis, MO, USA)- ≥98% (HPLC). The drug was dissolved in (99.9%) technical grade ethanol (V0T0041, Sigma Aldrich).

### 2.2. Molecular Docking Studies

#### 2.2.1. Protein Retrieval and Preparation

The Mdm2 p53 binding domain crystal structure was retrieved from the protein databank (https://www.rcsb.org/ (accessed on 19 December 2024)) with Pdb_Id: 1YCR [17]. This protein domain was used as a receptor and prepared to a suitable state for computational calculations using the protein preparation wizard from the Schrödinger suite. During this process, missing disulfide bonds, hydrogen atoms, side chains, and loops were added using Prime. Water molecules beyond 3 Å of the het groups were removed and the hydrogen bonds were optimized to avoid steric clashes. The structure was further refined through restrained minimization to an RMSD of 0.3 Å.

#### 2.2.2. Ligand Database Preparation

The ligand database—Zinc drug database (Zdd)—was downloaded from the ZINC12 library (https://zinc12.docking.org/browse/subsets/special, accessed on 1 February 2023) in canonical SMILES format. This database contains 2924 FDA-approved compounds which were all prepared to the lowest energy possible 3d configuration by varying tautomeric and ionization states at a targeted pH range of 7 ± 2 using Epik. This further generated multiple stereoisomers at a scale of up to 32 compounds per ligand of the original data set. These computations were executed using the OPLS4 force field. Additionally, the nutlin3a drug was downloaded from the PubChem (https://pubchem.ncbi.nlm.nih.gov/, accessed on 1 February 2023) database and prepared to a suitable 3d configuration state prior to docking studies. All computations were performed using the ligand preparation wizard in Schrödinger-Maestro v2021-2.

### 2.3. Search Space Mapping and Grid Generation

The docking search space grids were computed using the grid generation panel in Maestro from the Schrödinger suite. Here, the MDM2-p53 complex with Pdb_Id: 1YCR was used as an input and the grid computation performed at the centre of the p53’s transactivation domain. The outer grid box coordinates were set to 9.03, −14.57, and 24.22 in the x, y, and z axis, respectively, whilst the inner grid was kept at 10 angstroms (Å) for the same 3d axis. Both grids are very critical during docking, as they give a true measure of the effective search space and enable all ligands to find usual and asymmetric binding modes within the active site whilst confining their midpoints into a smaller box to save calculation time. The van der Waals radius scaling was set to 1 to soften the potential for nonpolar parts of the receptor, and the partial charge was set to 0.25.

### 2.4. Virtual Screening and Receptor-Based Ligand Docking

The entire database was screened and docked to the Mdm2 p53 binding domain through a flexible docking protocol employing the Glide standard precision (SP) algorithm in Maestro. The nature of docking simulations employed by this algorithm are the same as that of the High Throughput Virtual Screening (HTVS), except that HTVS reduces the number of intermediate conformations throughout the docking funnel and the thoroughness of the final torsional refinement and sampling. During the docking process, the Mdm2 p53BD structure was kept rigid (not even the hydroxyl and thiol groups could rotate), and flexibility was induced to all docking ligands. This was achieved through expansion of each input structure by generating variations on the ionization state, tautomer’s, stereochemistry, and ring conformations. Ultimately, the binding energies or affinities of all poses were analyzed to determine the best lead compounds.

### 2.5. Molecular Dynamics Simulations

To assess the binding specificity of the best lead compound, Ezetimibe, we subjected Ezetimibe-Mdm2 and nutlin3a-Mdm2 complexes to molecular dynamics (MD) simulations. We used the Desmond module in Maestro v2021-2 with the OPLS4 force field. We restricted the complexes by an orthorhombic box on which the TIP3P water solvation model was utilized [18,19]. The volume of the box was minimized to centre the complexes, and sodium ions (Na^+^) were added to counterbalance the overall charge on both systems. The Nose–Hoover thermostat and Martyna–Tobias-Klein barostat [18,20] techniques were used to maintain the temperature and pressure at 300 Kelvin and 1.01325 bar, respectively. We executed the simulations with the NPT ensemble class to ensure that the number of atoms, pressure, and timescales were kept constant. To simulate long-range electrostatic interactions, the Particle-Mesh-Ewald approach was used [21,22]. We also generated the RMSD and RMSF plots for Mdm2–Ezetimibe and Mdm2-nutlin3a complexes to understand the relative stability of the ligands in the receptor-binding pocket. Both simulations were monitored for 100 nanoseconds (ns), in which each had a 100 picosecond (ps) recording interval and generated 1000 frames. Finally, we used the MD trajectory analysis and simulation diagrams to analyze and present results.

### 2.6. In Silico Pharmacokinetics

ADMET (Absorption, Distribution, Metabolism and Toxicity) studies for Ezetimibe were conducted using SwissADME, ADMETSAR, and ProTox II [23]. We computed physicochemical properties such as molecular weight (MW), molecular refractivity (MR), the count of specific atomic types, and Polar surface area (PSA), employing the TPSA (Topological polar surface area) fragmental technique (which considers sulfur and phosphorus as polar atoms), lipophilicity, water solubility, pharmacokinetics, and drug-likeness.

### 2.7. Cell Lines and Cell Culture

The human malignant melanoma cells (A375), pancreatic cancer cells (PANC1), lung adenocarcinoma cell line (A549), human colorectal carcinoma cell line (HT-29), and human embryonic kidney cells (HEK293) listed in Table 1 were cultured in media containing 89% DMEM (Lonza Bioscience, Cambridge, MA, USA), 10% Fetal Bovine Serum (FBS) (Biowest, Nuaillé, France) and 1% penicillin-streptomycin (Biowest, Nuaillé, FranceUSA). Once confluent, the cells were washed with 1× phosphate-buffered saline (PBS) (Thermo Fisher Scientific, Waltham, MA, USA) three times and detached from T75 flasks by incubating cells with two millilitres Trypsin-EDTA (Thermo Fisher Scientific, Waltham, MA, USA) for 5–10 min at 37 °C. A measure of 2 mL DMEM (Lonza Bioscience, Cambridge, MA, USA) was added to the cells to stop the reaction of trypsin-EDTA. The cells were collected by centrifugation (1000× *g*) for 5 min and resuspended in fresh media for subsequent experiments.

### 2.8. Determination of Cell Viability by MTT Assay

The cancer cell lines (A375, A549, HT-29,PANC-1) and the normal cell line (HEK293) were plated in 96-well plates and with a seeding density of 20,000 cells per well. The cells were then treated with various concentrations of Ezetimibe for 48 h in three biological replicates. Following treatment, 10 µL of the MTT reagent was added and cells were incubated for a further 4 h at 37 °C. Dimethyl sulfoxide (DMSO) (D8418, Sigma Aldrich) was then added to the wells to solubilise the MTT crystals. The samples were incubated further for 15 min at 37 °C and absorbance readings were taken at 570 nM using the Varioskan^TM^ LUX multimode microplate reader (ThermoFisher Scientific, VL0000D0) and Varioskan software (v4.X). The percentage of cell viability was determined using the absorbance readings of treated and untreated cells, using the formula below. The IC_50_ values for each cell line were calculated using the AAT Bioquest IC_50_ online calculator [24].

$$\%Cell\;Viability=\frac{Absorbance\;(treated)}{Absorbance\;(untreated)}\times 100$$

### 2.9. Apoptosis Assay by Flow Cytometry

To determine the apoptotic effect of Ezetimibe on lung cancer (A549) and melanoma (A375) cell lines, apoptosis was assessed using the Annexin V/propidium iodide (PI) dual-staining method. Cells were seeded into 6-well plates at a density of 100,000 cells per well and treated with the IC50 concentrations of Ezetimibe for 48 h. For each treatment condition, three biological replicates were included. Post-treatment, cells were harvested by trypsinization, washed with 1× phosphate-buffered saline (PBS), and resuspended in 1× binding buffer. Subsequently, cells were stained with 5 µL of Annexin V-fluorescein isothiocyanate (FITC) and 5 µL of PI (BioLegend, USA) for 15 min at room temperature in the dark. After staining, samples were immediately analyzed using a BD FACS Aria III flow cytometer (BD Biosciences Franklin Lakes, NJ, USA). The percentages of apoptotic cells (early and late apoptosis) were calculated using FACS DIVA software (v10.0). Cells positive for Annexin V and negative for PI were considered early apoptotic, while cells positive for both Annexin V and PI were considered late apoptotic.

### 2.10. Cell Cycle Analysis by Flow Cytometry

#### 2.10.1. For A375 Cells

To investigate the effect of Ezetimibe on cell cycle progression in melanoma (A375) cells, flow cytometry was performed using propidium iodide (PI) staining. A375 cells were seeded into 6-well plates and grown to 80–90% confluence. For each treatment, three replicates were included. The cells were treated with 30 µM of Ezetimibe for 48 h. After treatment, both floating and adherent cells were collected by centrifugation and trypsinization, respectively. The cells were washed with 1× phosphate-buffered saline (PBS), fixed in 70% ethanol at −20 °C for 24 h, and stained with 500 µL of PI solution containing RNase A (0.1 mg/mL) and Triton X-100 (0.1%) for 30 min at 37 °C. The DNA content was analyzed using a BD FACS Aria III flow cytometer (BD Biosciences), and data were processed using FACS DIVA software (v10.0). Cell cycle phases (G0/G1, S, G2/M) were determined based on DNA content and represented as percentages.

#### 2.10.2. For A549 Cells

The impact of Ezetimibe on cell cycle progression in lung cancer (A549) cells was determined using the propidium iodide (PI) staining method and flow cytometry analysis. A549 cells were initially seeded in T25 cell culture flasks with 5 mL of culture media and grown to 80–90% confluence. The cells were then treated with the IC50 concentration of Ezetimibe (50 µM) for 48 h. After treatment, both floating and attached cells were collected by centrifugation and trypsinization, respectively. The cells were washed twice with 1× PBS to remove any residual culture media and other contaminants. Following this, the cells were stained with PI, a DNA intercalating dye, to assess cell cycle status. The DNA content was analyzed using a BD FACS Aria III flow cytometer (BD Biosciences), and data were processed using FlowJo software (v10.0). Cell cycle phases (G0/G1, S, G2/M) were determined based on DNA content and represented as percentages.

### 2.11. siRNA Mediated Knockdown of MDM2

Lung cancer (A549), melanoma (A375), colorectal cancer (HT-29), and human embryonic kidney (HEK293) cells were cultured in their respective optimal growth media under standard conditions (37 °C, 5% CO_2_) until 80–90% confluency. Cells were transfected with MDM2 siRNA (siMDM2) at varying concentrations (0 to 10 nM) using Lipofectamine 2000 (Thermo Fisher Scientific, Waltham, MA, USA) according to the manufacturer’s instructions. Transfections were carried out in 6-well plates, and cells were incubated with siRNA for 24 h to ensure adequate knockdown of Mdm2. Post-transfection, cells were treated with IC_50_ concentration of Ezetimibe specific to each cell line. For HT-29 and HEK293 cells, which lacked specific IC_50_ values, a standardized concentration of 50 µM Ezetimibe was used. After the treatment period, cell viability was assessed using the MTT assay. Briefly, MTT reagent was added to each well and incubated for 4 h at 37 °C. Formazan crystals formed by viable cells were solubilised in DMSO, and absorbance was measured at 570 nm using a microplate reader. Cell viability was expressed as a percentage relative to untreated controls.

### 2.12. Western Blot

#### 2.12.1. Treatment of Cell Lines for Protein Level Determination

Cells were cultured in T75 plates until the confluency of 80–90%. Cells were then subjected to treatment IC50 concentrations of Ezetimibe (50 µM for A549 and HEK293; 30 µM for A375) for 48 h. Following treatment, the media was transferred into 15 mL centrifuge tubes to collect the detached cells. The remaining attached cells were incubated with 2 mL of trypsin-EDTA for 10 min at 37 °C to detatch the cells. The detatched cells were transferred to respective centrifuge tubes containing the media collected from the same plates. The cells were then centrigfuged at 450× *g* for 5 min. The cells were washed with 1× PBS and centrifuged again to pellet the cells. The total protein was extracted from pelleted cells using the CeLytic M (C2978 Merck Millipore, MA, USA) mammalian cell lysis reagent. One ml of CelLytic M lysis reagent was added to each pellet, resuspended, and incubated for 15 min on a shaker. Following lysis, the protein samples were denatured through heating for 10 min at 95 °C. The lysed and denatured cells were centrifuged at 12,000× *g* for 15 min and the supernatant was transferred into fresh 1.5 mL tubes.

#### 2.12.2. Protein Concentration Determination

The protein concentrations were determined using the Qubit^TM^ Protein Broad Range (BR) Assay (A50668, Thermo Fisher Scientific, Waltham, MA, USA) and measured by spectrophotometry using a Qubit^TM^ Flex Fluorometer Qubit Fluorometer (Thermo Fisher Scientific, Waltham, MA, USA).

#### 2.12.3. Detection of p21, p53, and ERK1 Levels in Treated Cells Using Jess^TM^ Automated Western Blot System

The determination of protein expression levels of cell cycle related proteins (p21 and phosphorylated p53) following treatment in A375, A549, and HEK293 cells was conducted using the Jess^TM^ automated Western Blot system (ProteinSimple, Bio-techne, San Jose, CA, USA) and the 12–230 kDa Separation Module (SM-W001, ProteinSimple). Ten microlitres of protein samples were mixed with 2 µL of master mix and incubated at 95 °C for 5 min. Ten microlitres of protein and master mix samples were loaded on to a 25-well plate/module preloaded with buffers. Five microlitres of 1:50 anti-phospho-p53-ser15 (ab237514, abcam Cambridge, UK), 10 µL anti-ERK1 (ProteinSimple) and 1:50 anti-p21 (ab109420, abcam) primary antibodies were added to detect the phosphorylated p53 and p21 proteins. The 25-well chemiluminescence cartridge was used for the separation and immunodetection of proteins. The samples were run on the Jess automated immunodetection and quantification system (ProteinSimple, Bio-techne) for 3 h and data collection and analysis were performed using the Compass software version 6.2.0 (ProteinSimple, Bio-techne).

### 2.13. Statistical Analyses

All statistical analyses were performed using Prism GraphPad Software (v5.1) (San Diego, CA, USA). The cell viability data were expressed as the mean ± standard deviation (SD) from three independent experiments. The IC_50_ values for each cell line were calculated using the AAT Bioquest IC_50_ online calculator. The one sample T-test and Wilcoxon Signed Rank Test were used to determine the statistical difference between the cell viability percentage in treated and untreated cells. *p* values less than 0.05 were regarded as statistically significant.

## 3. Results

### 3.1. Molecular Docking of Ezetimibe and Nutlin3a to the Mdm2-p53 Binding Domain

**Figure 1:**
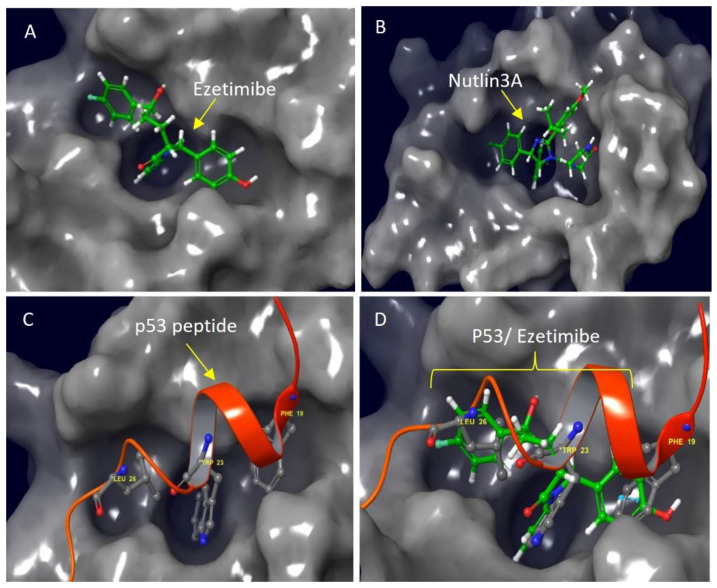
Molecular docking studies showing the most potent inhibitor of the Mdm2-p53 complex compared to nutlin3a. (**A**) The Ezetimibe molecule (green ball and stick carbon back-bone molecule with oxgen, nitrogen and hydrogens in red, blue and white respectively) bound into the Mdm2 hydrophobic cleft (grey surface). (**B**) The nutlin3a drug docked into the same Mdm2 pocket. (**C**) The p53 transactivation domain (red) also bound to the Mdm2 pocket (grey surface). The three critical residues (Leu26, Trp23 and Phe19) are also indicated in ball and stick models. (**D**) A graphical view of Ezetimibe’s inhibition activity and the accurate mode of binding that mimics p53 upon targeting the Mdm2’s hydrophobic cleft.

The molecular docking studies show that Ezetimibe binds to the same binding pocket of Mdm2 as the nutlins. The chemical name for Ezetimibe is 1-(4- fluorophenyl)-3(R)-[3-(4-fluorophenyl)-3(S)-hydroxypropyl]-4(S)-(4-hydroxyphenyl)-2-azetidinone. It belongs to the azetidinone class of compounds which are characterized by a β-lactam ring and a grey surface representation of Ezetimibe and its superposition with p53 is shown in Figure 1A,D.

Nutlins are spirooxindole compounds which were developed as non-peptidic mimics of the p53 peptide that binds to Mdm2. Based on our docking studies, Ezetimibe binds snugly into the same binding site as nutlin3a, which is now in clinical trials for the treatment of cancer. Therefore, this study revealed Ezetimibe as a highly effective inhibitor upon targeting the Mdm2-p53 binding domain, with a docking score of −7.919 Kcal/mol (Table 2). Notably, this binding energy was better than the −6.359 Kcal/mol observed in the case of nultin3a (Table 2).

**Figure 2:**
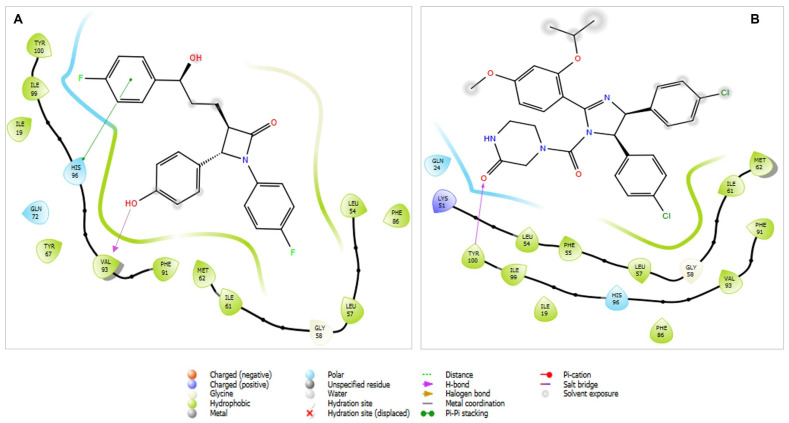
Two-dimensional ligand interaction diagrams of Ezetimibe and nutlin3a upon the Mdm2-p53 binding domain. (**A**) A 2D Interaction of Mdm2–Ezetimibe complex in which the hydrophobic and polar residues dominate the binding coordination. (**B**) A 2D Interaction of MDM2-nutlin3A complex in which the hydrophobic, polar and a single positively charged residue dominate the binding coordination. Note: both diagrams were taken at 4 Å axis around the compounds within the active site.

Furthermore, the active site is also dominated by a mixture of highly hydrophobic and polar residues (Figure 1 and Figure 2), and both drugs make multiple hydrophobic and polar interactions to facilitate the binding coordination. Primarily, three hydrophobic pockets in Mdm2, defined by critical side chains on the p53 peptide, namely Phe19, Trp23, and, Leu26, form the main anchor of the Mdm2-p53 interaction [25]. Moreover, a superimposition of Ezetimibe to the p53 peptide in the context of the Mdm2 binding site shows a good alignment, indicating that Ezetimibe may be a better competitive inhibitor for p53 (Figure 1C,D) than the nutlins.

Importantly, Ezetimibe fits well in the three binding pockets in which the glucuronidation site is positioned to the Phe19 pocket, whilst the hydrogen bond it forms with Val93 is in the Trp23 pocket. This hydrogen bond that Ezetimibe forms is interesting because Trp23 in the p53 peptide also forms a hydrogen bond with this residue and this interaction has been reported to be crucial for p53-Mdm2 binding [26]. Additionally, Ezetimibe also forms a π–π stacking interaction with His96, which should augment the binding energy. The binding pocket induced by Ezetimibe is much smaller compared to the bulky nutlin3a-induced pocket (Figure 2). The topological arrangement described here would be sterically bulky for the glucuronidated version which binds to the Niemann-Pick C1-Like 1 receptor. Altogether, these features suggest that only the parent drug would play the competitive role with p53.

### 3.2. Molecular Dynamics and Molecular Mechanics—Generalized Born Surface Area (MM-GBSA) Studies of Ezetimibe and Nutlin3a in the Mdm2 Hydrophobic Pocket

**Figure 3:**
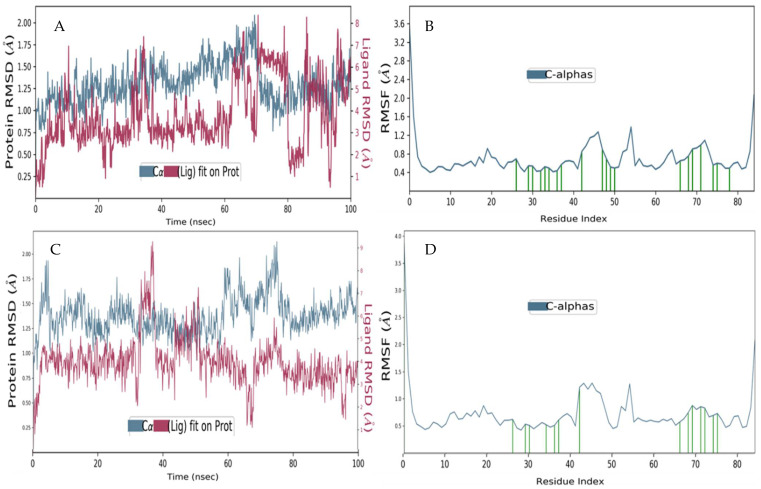
Molecular dynamic studies of Mdm2 complexed with Ezetimibe and nutlin3a. (**A**) Scalar distance of the Mdm2–Ezetimibe complex over a 100 ns simulation time (The P-L RMSD) and (**B**) characterization of the local protein fluctuation (The P_RMSF), with Ezetimibe interacting residues shown as green vertical lines. (**C**) Scalar distance of the MDM2-nutlin3A complex over a 100 ns simulation time (The P-L RMSD) and (**D**) characterization of the local fluctuation of the protein (the P_RMSF), with nutlin3a interacting residues shown as green vertical lines. Note: RMSD stands for Root Mean Square Deviation, and RMSF stands for Root Mean Square Fluctuation.

We conducted simulation studies on Ezetimibe binding to the Mdm2 hydrophobic pocket to understand the configurational adaptability during the molecular recognition process. For comparison, we performed the same simulations on nutlin3a bound to Mdm2. Throughout the simulation, the Mdm2–Ezetimibe interactions were observed for 100 ns. The RMSD for Ezetimibe depicts a consistent interaction pattern with the Cα atoms of the Mdm2 receptor (Figure 3A,B). In contrast, the profile for nutlin3a suggests that it has little resident time in the binding pocket. It was observed that from approximately 0 to 30 ns and 50–100 ns, the nutlin3a drug candidate was completely not in contact with the Cα atoms of the Mdm2 protein (Figure 3C). This is significant because it shows that nutlin3a was dissociated from the complex for about 80% (~>80 ns) of the simulation period (Figure 3). In contrast, there are persistent interactions between Ezetimibe and Mdm2 throughout the simulation period. Potentially, therefore, Ezetimibe could be a better antagonist of the Mdm2-p53 interaction compared to nutlin3a. However, the RMSD data suggests that further lead optimization on the Ezetimibe scaffold may be necessary to stabilize the compound even more into the Mdm2 pocket, thus attaining an even better interaction. The RMSF data also depict an excellent trend in the case of Ezetimibe, with all interacting residues (indicated in green vertical lines) in their lowest energy state, with an average of about 0.4Å (Figure 3B). This occurs partially on nutlin3a which averages at about 0.7Å, with some residues reaching above 1.3Å. It is also worth noting that Ezetimibe is stabilized by more residues (19), whilst nutlin3a interacts with fewer residues (14). Taken together, all these properties lead to compromised thermodynamic stability of the Mdm2-nutlin3a complex (Figure 3C,D). Notably, the RMSF data further reinforces that the Mdm2–Ezetimibe complex is more thermodynamically favourable than Mdm2-nutlin3a.

The binding free energy (Δ*G*_binding_) of the protein–ligand complexes was calculated using the Prime MM-GBSA module in the Schrödinger suite. The protein-ligand complexes were first prepared using the Protein Preparation Wizard, as described above. Ligands were prepared using LigPrep to ensure proper ionization states, tautomers, and 3D geometries. For molecular dynamics (MD) simulations, representative snapshots were extracted at 10-frame intervals from a 100 ns trajectory generated in Desmond. The MM-GBSA calculations were conducted using the VSGB 2.0 [27] solvation model and the OPLS4 force field. The binding energy was computed as Δ*G*_binding_ = *G*_complex_ − (*G*_receptor_ + *G*_ligand_), where *G*_complex_, *G*_receptor_, and *G*_ligand_ represent the total energies of the bound complex, the unbound receptor, and the unbound ligand, respectively.

Energy contributions, including van der Waals, electrostatic, and solvation energies, were analyzed to evaluate the protein–ligand interactions. The entropy contribution (−*T*Δ*S*) was not directly included but can be considered for future analyses using quasiharmonic or normal mode approaches. In this study, the average free energy of binding was obtained to be −62.31 kcal/mol and −52.56 kcal/mol for both Mdm2–Ezetimibe and Mdm2-nutlin3a complexes, respectively. These results further highlight Ezetimibe as the better antagonist of the Mdm2-p53 complex, which underscores its more promising cancer-therapeutic potential compared to nutlin3a.

### 3.3. Binding Interaction Analysis

**Figure 4:**
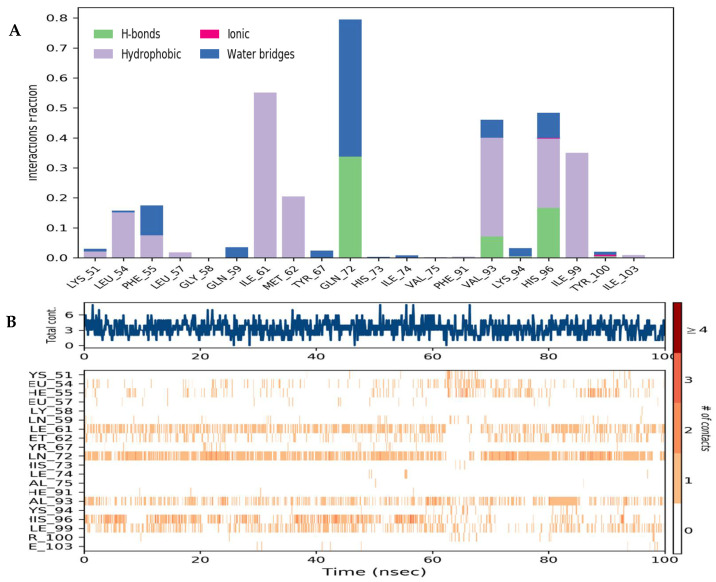
Protein–ligand contacts of the Mdm2–Ezetimibe complex. (**A**) The interaction fraction per residue of the MDM2–Ezetimibe complex in which the hydrophobic and water bridge interactions dominate the binding coordination. (**B**) The extent of binding as well as the number of contacts made throughout the simulation.

To analyze the thermodynamic profile of the Ezetimibe-Mdm2 interaction within the Mdm2 pocket, we probed the binding coordination formed by Ezetimibe and compared it with that of nutlin3a. This analysis investigated four interactions—hydrogen bonding, hydrophobic interactions, ionic bonds, and salt bridges (Figure 4). Both drugs showed no ionic bond interactions with the binding pocket. However, hydrogen bonds, hydrophobic, and water bridge interactions facilitate the binding interactions in both complexes. Notably, Ezetimibe interactions are dominated by hydrophobic forces and water bridges (Figure 4A). Additionally, Ile61, Gln72, Val93, His96, and Ile99 are the five most critical residues facilitating the binding coordination of the complex (Figure 4B). Most importantly, the interaction fraction of Mdm2 is very low towards nutlin3a in most of the interacting residues such as Phe55, Val93, and Thr100.

**Figure 5:**
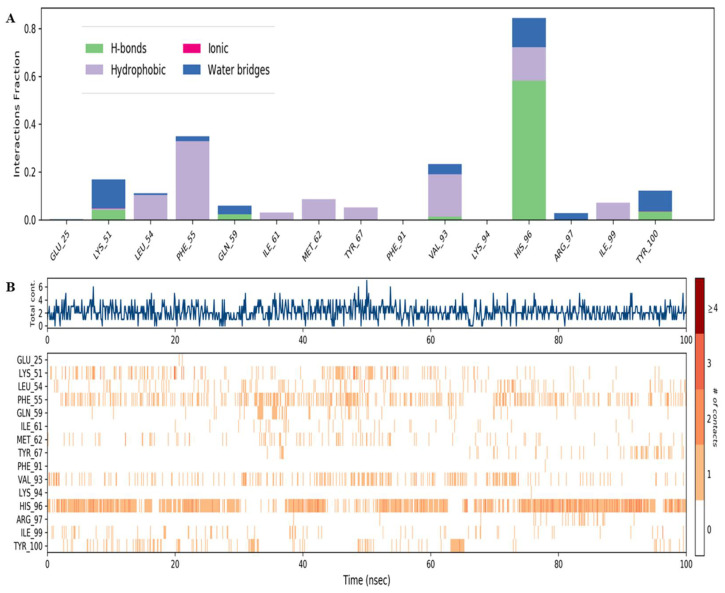
Protein–ligand contacts of the Mdm2-nutlin3a complex. (**A**) Interaction of Mdm2-nutlin3a complex in which the hydrophobic and water bridge interactions dominate the binding coordination. (**B**) The extent of binding as well as the number of contacts made throughout the simulation.

Nutlin3a also showed that hydrophobic interactions and water bridges dominate the binding interactions it exhibits towards the Mdm2 receptor, and His99, Phe55, and Val93 are the only critical residues facilitating the binding coordination (Figure 5A,B). However, it is worth noting that the points of contact on the Mdm2-nutlin3A complex are not persistent throughout the simulation, as depicted in Figure 5B. This observation further confirms that nutlin3a exhibits a lower drug resident time and may be frequently displaced during the simulation.

### 3.4. The Pharmacokinetic Properties of Ezetimibe and Nutlin3a

This in silico study of Ezetimibe pharmacokinetics was conducted as described in the “methods” and the Ezetimibe profile was compared with nutlin3a (Table 2) to evaluate the drug-likeness of Ezetimibe. Ezetimibe has a single rule of five violation whereas nutlin3a has four. It has a lower molecular weight of 409,433 g/mol compared to nutlin3a (581,494 g/mol). Furthermore, Ezetimibe and nutlin3a have comparable lipophilicity, with cLogP values of 4.33 and 4.56, respectively. However, due to pre-systemic metabolism, they have poor oral bioavailability. A plausible therapeutic inhibitor of Mdm2 should have the following desirable properties: (a) a high binding affinity and specificity, (b) potent cellular activity in cancer cells with wild-type p53, and (c) a highly desirable pharmacokinetic profile [28]. Ezetimibe has satisfactory properties and deserves investigation as an anticancer agent.

### 3.5. Comparative Docking Study of Ezetimibe with Other Newly Developed MDM2 Inhibitors

**Figure 6:**
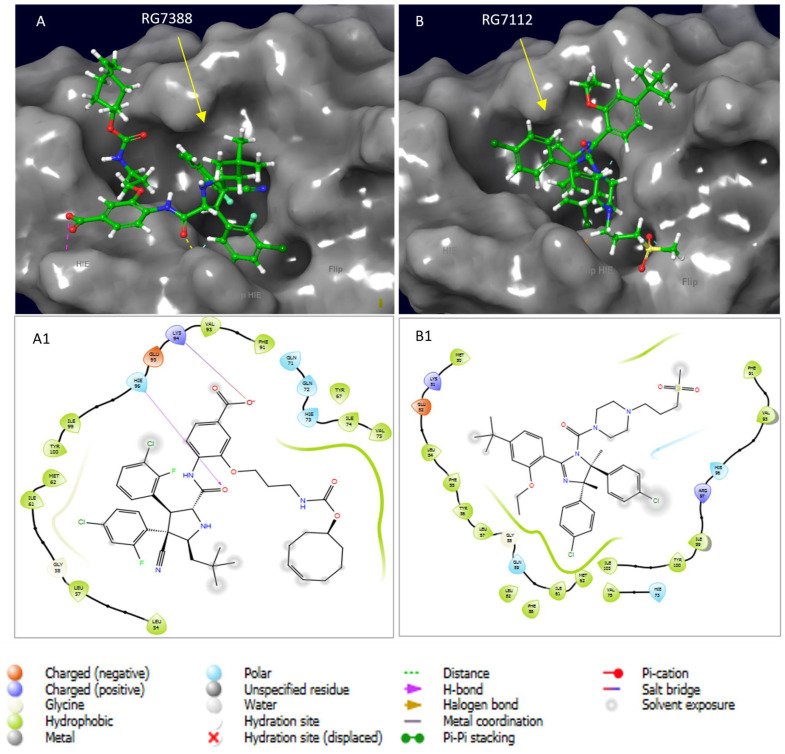
A comparative docking study showing the two newly developed inhibitors of the Mdm2-p53 complex—RG7112 and RG7388. (**A**,**B**) The RG7112 and RG7388 molecule (green) bound into the Mdm2 hydrophobic cleft (grey surface). (**A1**,**B1**) A 2D interaction diagram of Mdm2-RG7112/RG7388 complexes in which the hydrophobic, polar, and charged residues dominate the binding coordination. Both diagrams were taken at 4 Å axis around the compounds within the active site. The red, blue, green and yellow atoms represent oxgen, nitrogen, halogens (chlorine and florine) and sulfur respectively.

A further comparative docking study of Ezetimibe and the new MDM2 inhibitors—RG7388 and RG7112—was conducted (Figure 6). This approach allowed us to determine the therapeutic potential of Ezetimibe, since both RG7388 and RG7112 have shown significant strides in clinical trials [29,30]. It is worth mentioning that the docking scores for these two inhibitors are −6.337 kcal/mol and −6.222 kcal/mol, respectively. This is critical because it implies that Ezetimibe exhibits the highest binding energy and is a better disruptor of the Mdm2-p53 complex, with a docking score of −7.919 kcal/mol. Furthermore, it is worth noting that both compounds are much bulkier than Ezetimibe, which increases the chance of steric clashes and making it difficult to find a better binding mode within the p53 hydrophobic pocket. Therefore, our results do not negate the potential therapeutic effect of RG7388 and RG7112. However, we present Ezetimibe as a better alternative disruptor of the Mdm2-p53 complex that can achieve better efficacy than most of the currently known inhibitors.

### 3.6. Ezetimibe Is a Potential Anticancer Drug Targeting the p53/Mdm2/RBBP6 Complex

Currently, Ezetimibe is given orally to treat cholesterolemia. It is activated in the intestines into the pharmacologically active Ezetimibe glucuronide. The results from the current study indicate that the parent drug binds to Mdm2, the prototypical negative regulator of p53. This study shows that Ezetimibe is toxic to specific cancer cell lines, such as A375 and A549. It is not toxic to a normal human embryonic kidney cell line at the same concentrations. Therefore, Ezetimibe may be a candidate for anticancer therapy when given parenterally to bypass pre-systemic metabolism.

### 3.7. Ezetimibe Toxicity to Cancer Cell Lines

**Figure 7:**
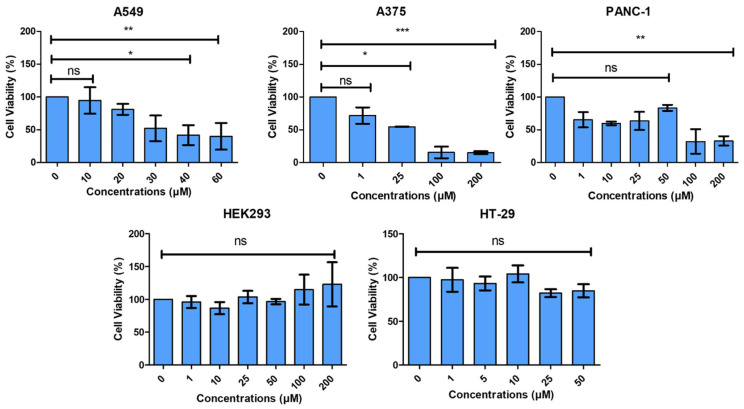
Selective cytotoxicity of ezetimibe. MTT assays were conducted on Ezetimibe-treated A549, A375, PANC-1, HEK293, and HT-29). The experiments were conducted in triplicate. Each bar represents the mean cell viability percentage relative to control (untreated or 0) and error bars indicate the standard deviation of at least three independent experiments. Statistical significance was evaluated using a one-way ANOVA. *p*-value * *p* < 0.05, ** *p* < 0.01, *** *p* < 0.001, and ns (non significant).

We tested the toxicity of Ezetimibe to several human cancer lines, including the human melanoma cell line, A375, the human pancreatic cell line (PANC-1), the breast cancer cell line (MCF7), the lung cancer cell line (A549), the colorectal cell line (HT-29), and the normal human embryonic kidney cell line (HEK293) using the MTT assay. Ezetimibe strongly inhibits the growth of the melanoma (A375) and lung cancer (A549) cell lines, with IC_50_ concentrations of 30.7 µM and 48.34 µM, respectively, compared to the lung cancer cell line and the pancreatic cancer cell line (Figure 7). At these concentrations, Ezetimibe does not exhibit toxicity to the normal human embryonic kidney cell line (HEK293), breast cancer cells (MCF7), and the p53 negative colorectal cell line (HT-29). Interestingly, previous studies showed that Mdm2 and Mdm4 are highly expressed in melanoma [31,32]. Ezetimibe inhibits approximately 20% of the melanoma cell line at the lowest concentrations tested (1 µM) and more than 80% at highest concentration (200 µM).

Notably, HT-29 cells are characterized by the overproduction of the p53 tumour antigen, harbouring a mutation at position 273, where arginine is replaced by histidine [33]. Studies have shown that this mutation in the *Tp53* gene significantly influences cancer cell responses to treatment. HT-29 cells, carrying the mutant p53, exhibit heightened resistance to the cytotoxic actions of certain drugs compared to cell lines with wild-type p53 [33]. This resistance mechanism has also been observed in PANC-1 cells, which share the same mutation at position 273, notably affecting drug responses within the p53 pathway [34,35,36].

**Figure 8:**
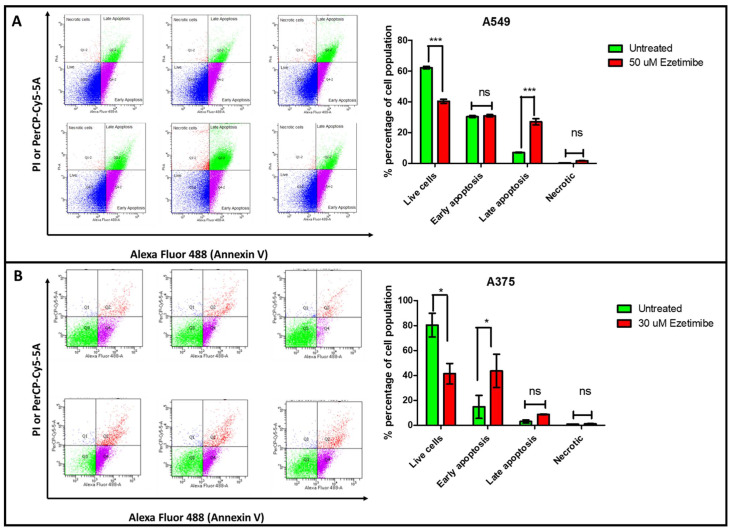
Flow cytometric analysis of apoptosis and necrosis in (**A**) A549 and (**B**) A375 cells following treatment with IC_50_ concentrations of Ezetimibe. Cells were stained with Annexin V/PI and analyzed using flow cytometry to quantify live, early apoptotic, late apoptotic, and necrotic cell populations. Bar graphs show the percentage of cell populations in each category. Statistical significance was evaluated using a one-way ANOVA. *p*-value * *p* < 0.05, *** *p* < 0.001, and ns (non significant).

In the context of Ezetimibe treatment, it was observed that the mutant p53 in HT-29 and PANC-1 cells impacted their response differently compared to other cell lines tested. Despite the documented cytotoxicity of Ezetimibe in A549 and A375 cancer cell lines, PANC-1 displayed cell death at concentrations above 100 µM, while HT-29 cells showed no signs of cytotoxicity across all concentrations. This unique behavior emphasizes the complex interplay between Ezetimibe and the altered p53 pathway in HT-29 and PANC-1 cells. It appears that Ezetimibe causes an arrest of cells at the G1 phase of the cell cycle (Figure 8).

### 3.8. Ezetimibe Induces Apoptosis in A549 and A375 Cancer Cells

We assessed the ability of Ezetimibe to induce apoptosis in lung cancer (A549) and melanoma (A375) cell lines using Annexin V and propidium iodide (PI) staining, followed by flow cytometry. The flow cytometry analysis shows an increase in apoptotic cells in Ezetimibe-treated A549 cells compared to untreated controls, as shown in Figure 8A. In treated A549 cells, a substantial population shifted towards early and late apoptosis quadrants, indicating that Ezetimibe triggers significant apoptosis in lung cancer cells.

Similarly, in the melanoma cell line (A375), Ezetimibe treatment resulted in a marked increase in apoptotic cells, as shown in Figure 8B. The flow cytometry results display a considerable population of A375 cells transitioning to early and late apoptosis compared to untreated controls. These results indicate that Ezetimibe induces apoptosis in both lung cancer and melanoma cells.

### 3.9. Ezetimibe Induces G1 Cell Cycle Arrest

**Figure 9:**
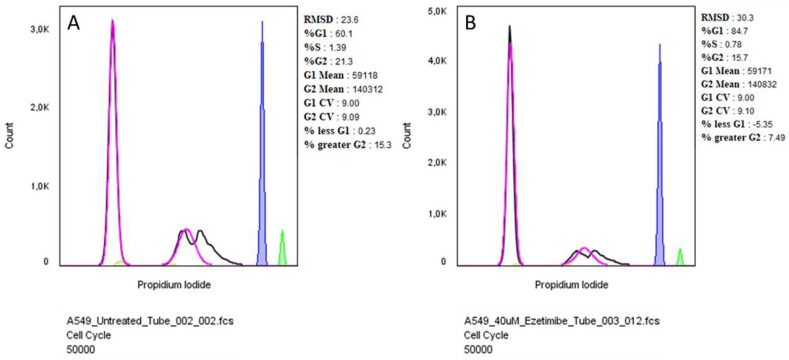
Cell cycle analysis of A549 cells following treatment with Ezetimibe. Cells were stained with propidium iodide and analyzed using a BD FACS Aria III flow cytometer and FLowJo Software (v10.10). Panel (**A**) shows the cell cycle distribution of untreated A549 cells, while panel (**B**) depicts A549 cells treated with 40 µM Ezetimibe. The x-axis represents DNA content, as measured by propidium iodide fluorescence intensity, and the y-axis shows the number of cells. The blue graph represents the G1 phase, whilst the green represents the G2/M phase. The pink and black graphs represent the overall distribution of cells. The percentage of cells in the G1, S, and G2 phases of the cell cycle is indicated for each panel.

Cell cycle analysis of A549 (human lung adenocarcinoma) and A375 (human melanoma) cell lines demonstrates that treatment with Ezetimibe induces significant cell cycle arrest at the G1 phase. As shown in Figure 9, untreated A549 cells displayed a typical cell cycle distribution, with 60.1% of cells in the G0/G1 phase, 1.39% in the S phase, and 21.3% in the G2/M phase. However, upon treatment with 40 µM Ezetimibe, 84.7% of the cells were arrested in the G0/G1 phase, while the population of cells in the G2/M phase was reduced to 15.7%. This indicates that Ezetimibe effectively halts the progression of A549 cells beyond the G1 phase, without causing a substantial change in the S-phase population.

**Figure 10:**
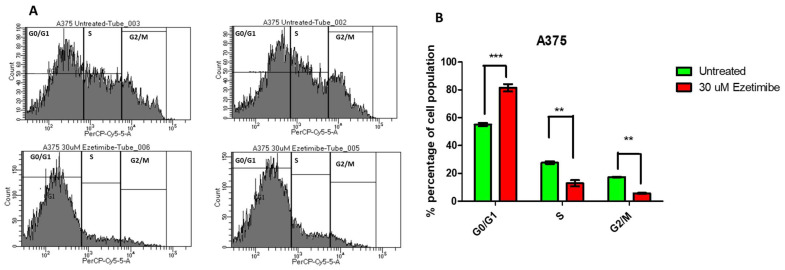
Cell cycle analysis of A375 cells following treatment with Ezetimibe. Cells were stained with propidium iodide and analyzed using flow cytometry and FACS DIVA software (V10.0). Panel (**A**) shows the cell cycle distribution of untreated A375 cells, while panel (**B**) depicts A375 cells treated with 30 µM Ezetimibe. The x-axis represents the DNA content, as measured by PerCP-Cy5-5-A fluorescence intensity, and the y-axis indicates the number of cells. The histograms highlight distinct cell cycle phases. The bar graph compares the percentage of cells in G0/G1, S, and G2/M phases between untreated and treated cells. Statistical significance was evaluated using a one-way ANOVA*. p*-value ** *p* < 0.01, *** p < 0.001.

Similarly, in the A375 melanoma cell line, untreated cells showed a normal cell cycle distribution, with significant proportions of cells in the G0/G1 and G2/M phases. After treatment with 30 µM Ezetimibe, the proportion of cells in the G0/G1 phase increased significantly, indicating a clear G1 arrest, while cells in the G2/M phase decreased markedly, as shown in Figure 10. The S phase population remained relatively unchanged, suggesting that Ezetimibe specifically induces G1 arrest without affecting DNA synthesis during the S phase.

In both cell lines, Ezetimibe treatment results in a pronounced accumulation of cells in the G0/G1 phase and a corresponding reduction in the G2/M phase population. These findings strongly suggest that Ezetimibe induces cell cycle arrest at the G1 phase.

### 3.10. Ezetimibe Treatment Downregulates the p53/p21 Axis and ERK1 in Lung and Melanoma Cells

**Figure 11:**
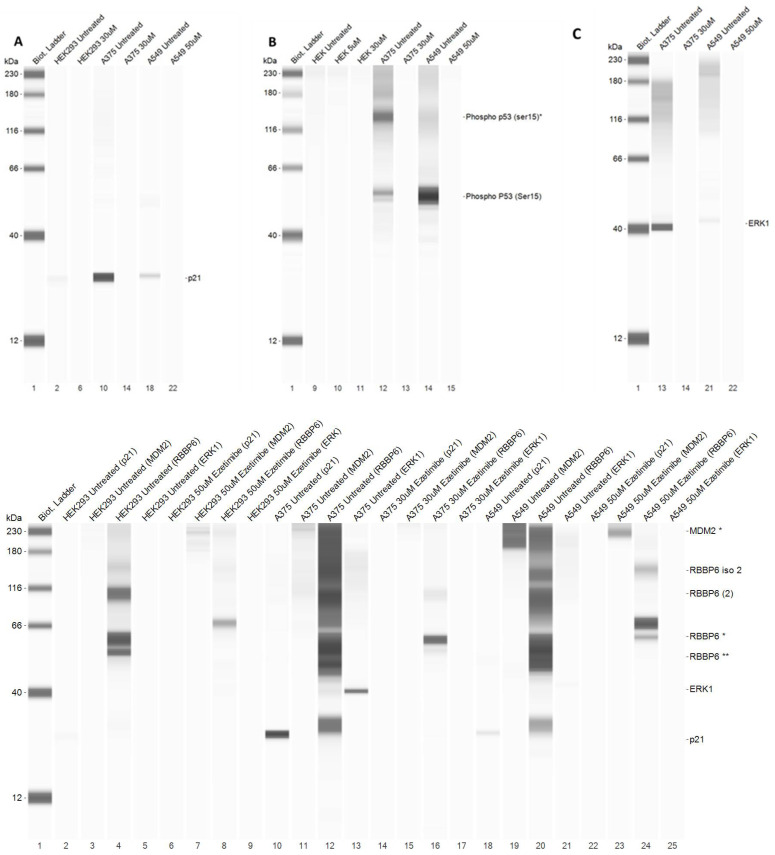
Western blot analysis illustrating the effects of Ezetimibe on the expression levels of p21, phosphorylated p53 (Ser15), and ERK1 in HEK293, A549, and A375 cell lines. Panel (**A**) shows a reduction in p21 levels in A375 and A549 cells following Ezetimibe treatment. Panel (**B**) demonstrates decreased phosphorylation of p53 at Ser15, indicating downregulation of p53 activation. Panel (**C**) highlights the significant reduction in ERK1 expression in both A375 and A549 cells after Ezetimibe exposure.

To assess the molecular mechanism underpinning cancer cell death after Ezetimibe treatment, we tested the p53 pathway and ERK1 expression in the melanoma cell line, A375, and the lung cancer cell line, A549. We compared this to untreated cells and the normal cell line, HEK293 (Figure 11). The untreated HEK293, A549, and A375 cell lines displayed varying amounts of p21, with HEK293 cells exhibiting comparatively lower levels (Figure 11A). The untreated A375 cell line showed the highest concentration of p21. Intriguingly, Ezetimibe-treated cell lines showed no detectable p21 expression, suggesting that Ezetimibe treatment downregulated the p53 pathway.

Phosphorylated p53, with an apparent size of 53 KDa, was exclusively detected in untreated A549 and A375 cell lines (Figure 11B), but remained undetected in Ezetimibe-treated cells, mirroring the pattern observed with p21 expression. Phosphorylated p53 was not detected in treated and untreated HEK cells and was downregulated in treated cancer cells. Interestingly, the phospho p53 antibody revealed a second and prominent band at approximately 130 KDa in A375 cells, adding complexity to the p53 expression pattern, which warrants further investigation.

ERK1, with a size of 44 kDa, was prominently detected in untreated A375 and A549 cell lines (Figure 11C) but was significantly downregulated following Ezetimibe treatment in both cell lines, mirroring the reduction observed in the p53/p21 axis. In contrast, untreated and treated HEK cells showed no substantial changes in ERK1 expression, suggesting a specific effect of Ezetimibe on cancer cells. The downregulation of ERK1 in treated A375 and A549 cells highlights the potential involvement of the MAPK/ERK signaling pathway. These findings collectively indicate that Ezetimibe exerts its anticancer effects by downregulating both the p53/p21 axis and ERK1, leading to cell cycle arrest and reduced proliferation in A549 and A375 cells.

### 3.11. The Dependence of Ezetimibe Activity on Mdm2 Expression

**Figure 12:**
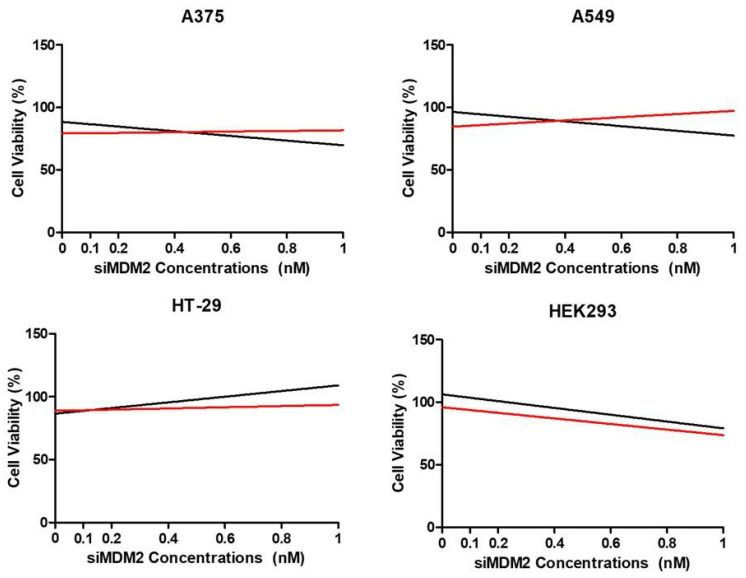
Ezetimibe dependence on Mdm2. Cell viability of A549, A375, HT-29, and HEK293 cells was investigated after treatment with siMDM2 alone (black) and combined with Ezetimibe at IC50 concentrations (red). The x-axis represents the concentrations of siMDM2 (0 to 10 nM) and the y-axis shows the percentage of cell viability relative to untreated controls. Cell viability was determined using the MTT assay, and all experiments were performed in triplicate. The graph shows the best fit depicting the trends during the experiment. The raw data are presented in the Supplementary File, Figure S1A.

To test whether Ezetimibe activity depends on Mdm2, we measured the cell viability of Ezetimibe-treated cells when Mdm2 was knocked down by siRNA. We find that Ezetimibe treatment tends to rescue cell death caused by Mdm2 knockdown in A375 and A549 cells but not in the HEK293 and HT29 cells (Figure 12). HT29 cells with arginine 273 to histidine change retain the wild-type conformation but lack the transactivation and growth suppression capabilities. Mdm2 knockdown in these cells appears to promote proliferation. As expected, in HEK293 cells, the depletion of Mdm2 increased the cell death rate. These results suggest that Ezetimibe perturbs Mdm2 function.

## 4. Discussion

Ezetimibe, a cholesterol absorption inhibitor when administered orally, possesses unique properties that make it a promising anticancer therapeutic drug. It targets the p53/Mdm2 complex when given parenterally. This distinct dual functionality sets it apart from other drugs. Ezetimibe monotherapy is recommended for the treatment of primary hypercholesterolemia in adults, but it is also co-administered with statins under certain conditions. While Ezetimibe’s success in treating cardiovascular diseases is well-documented, anecdotal reports suggest it may also have an anticancer therapeutic effect. However, the proposed mechanism for this effect is presumed to be cholesterol reduction. Our study reports that Ezetimibe interferes with Mdm2 function and causes the selective death of cancer cell lines, probably dependent on their p53 status. We discuss the plausibility of Ezetimibe as an Mdm2-binding drug and the molecular pathways involved in its anticancer activity.

Molecular docking studies have revealed that Ezetimibe binds effectively to the p53 binding domain of Mdm2, forming a more thermodynamically stable complex than nutlin3a, a known Mmd2 inhibitor. This solid and stable binding affinity, a key factor in drug effectiveness, suggests that Ezetimibe can potentially disrupt the Mmd2-p53 interaction. Molecular dynamics simulations further confirm the stability of the Ezetimibe-Mdm2 complex, showing consistent interactions over a 50-nanosecond simulation period, unlike the transient interactions observed with nutlin3a. This stability significantly enhances the confidence in Ezetimibe’s potential as an Mdm2-binding drug.

Functionally, Ezetimibe induced significant cytotoxicity in A549 and A375 cancer cell lines at IC_50_ concentrations of 48.34 µM and 30.7 µM, respectively, without affecting the viability of HEK293 cells. This selective toxicity highlights its potential as a cancer therapeutic, with minimal adverse effects on normal cells. Other studies have also tested the impact of Ezetimibe on breast cancer cell lines, with evidence showing toxicity to both MCF7 (wild-type p53) and T47D (mutant p53) cell lines. Similar effects were observed in triple-negative breast cancer cell lines (MDA-MB-231) and mouse-derived breast cancer cell lines (4T1), where Ezetimibe inhibited proliferation and induced cell death [8]. Another study by Zheng et al. (2023) found that Ezetimibe promoted the death of HCT116 and CaCO2 cell lines by activating the mTOR signaling pathway [7].

Interestingly, HT-29 and PANC-1 cells harboring a mutant p53, exhibited resistance to Ezetimibe. The correlation between p53 status and drug sensitivity is well-documented. For instance, studies from the National Cancer Institute (NCI) anticancer drug screen demonstrated that cells with mutant p53 exhibited less growth inhibition when treated with most clinically used anticancer agents compared to wild-type p53 cell lines [37]. Additionally, other studies emphasize the crucial role of the Mdm2-p53 interaction in cancer, with Mdm2 and Mdm4 (MDMX) regulating p53 activity and stability, leading to p53 inactivation in various cancers [38,39,40].

Cell cycle analysis showed that Ezetimibe treatment leads to G1-phase cell cycle arrest in A549 and A375 cells, accompanied by a decrease in the G2/M phase population. Similarly, previous studies by Qin et al. (2014) demonstrated that Ezetimibe inhibits the proliferation of vascular smooth muscle cells (VSMCs) by inducing cell cycle arrest at the G0/G1 phases [41]. Additionally, recent studies by He et al. (2023), observed that Ezetimibe significantly increases the G1 phase population in triple-negative breast cancer cell lines (MDA-MB-231 and 4T1) in a dose-dependent manner after 48 h of treatment [8].

Western blot analyses demonstrated that Ezetimibe treatment resulted in the downregulation of p21 and phosphorylated p53 in A375 and A549 cells, suggesting a disruption of the p53 signalling pathway. Phosphorylated p53 at Ser15 plays a crucial role in the activation of p53 in response to DNA damage, leading to cell cycle arrest or apoptosis. Interestingly, no phosphorylated p53 at Ser15 was detected in Ezetimibe-treated cancer cell lines, although it was present in untreated A549 and A375 cell lines. A study by Li and colleagues showed that treatment of rat VSMC cells with Ezetimibe resulted in the downregulation of cyclin D1, CDK4, pRb, E2F, and extracellular signal-regulated kinase (ERK1) proteins [41]. In the current study, Ezetimibe probably downregulates this pathway because the expression of ERK1 is depleted in Ezetimibe-treated A375 and A549 cells. This pathway plays a critical role during the G1/S transition.

The knockdown of Mdm2 causes a decline in cell proliferation in A375 and A549 cells, which is rescued by Ezetimibe treatment. This suggests that Ezetimibe is inactive in the absence of Mdm2. This reinforces the in silico modeling study showing a strong affinity between Ezetimibe and the p53 binding site on Mdm2. Taken together, these results indicate that Ezetimibe selectively kills cancer cells in the context of a deregulated p53/p21 pathway.

Currently, Ezetimibe is administered as a prodrug, which is activated in the brush border into an active Ezetimibe glucuronide. Our in silico data show that Ezetimibe binds into the MDM2-p53 binding domain, remarkably mimicking the p53 N-terminal peptide. While Ezetimibe does not structurally resemble nutlins, it accurately mimics p53 binding to the Mdm2 hydrophobic cleft based on molecular docking simulations. Ezetimibe, however, has structural vulnerabilities that hinder its use as an anticancer agent, such as its conversion by metabolic enzymes in the intestines. This results in the negligible bioavailability of Ezetimibe, which is unsuitable for use in the treatment of cancer by oral administration. Nonetheless, the evidence suggests that Ezetimibe may be an effective anticancer drug when administered parenterally. This underscores its potential in the field of oncology. Its selective toxicity towards cancer cells, coupled with its ability to disrupt critical cell cycle and apoptosis pathways, further underscores the therapeutic potential of Ezetimibe.

## 5. Conclusions

In its current application, Ezetimibe is a prodrug used to treat cholesterol by inhibiting its uptake, primarily from the brush border in the small intestine. In this environment, Ezetimibe is converted to glucuronidated form. It has been assumed that Ezetimibe’s anticancer activity was due to reduced cholesterol uptake and unclear subcellular interactions. The present article reports solid evidence showing that Ezetimibe in the parent form binds to the p53 binding site on Mdm2 and that Mdm2 may influence the efficacy based on the siRNA experiments. The current evidence, therefore, suggests that Ezetimibe, in its parent form, may be used as an anticancer drug when given parenterally, as it is vulnerable to pre-systemic metabolism. This mechanism of action would not function if Ezetimibe were given orally. This is the first report of Ezetimibe as a potential injectable anticancer treatment based on laboratory experiments. We have reported this in previous work based on in silico studies, demonstrating the thoroughness and reliability of our findings and instilling confidence in the validity of our research.

In the present study, Ezetimibe appears to interfere with ERK1 signaling and disrupt the transition from G1. Moreover, Ezetimibe reverses the activation of the p53/p21 pathway. Our study is consistent with the previous research mentioned in the introduction, which reported that Ezetimibe blocked the cell cycle at the G1 stage in a breast cancer cell line. This study reports that Ezetimibe activated the PGGFRb/Akt signaling pathway. Interestingly, PGGFR signals via RAS and activates ERK as an effector transcription factor.

Our study raises important questions about the role of Mdm2 in Ezetimibe’s anticancer activity. While we have shown that Ezetimibe’s effectiveness is reduced in depleted Mdm2, it remains to be elucidated if the overexpression of Mdm2 is mandatory for the Ezetimibe-dependent killing of cancer cells. These findings open new avenues for further research and could potentially lead to the development of more effective cancer treatments. We are conducting a preclinical study that will provide additional insights into these critical aspects of the drug’s mechanism of action, paving the way for future clinical applications.

## Figures and Tables

**Table 1:** List of cell lines and their p53 statuses.

Cell Line	Description (Full Name)	P53 Status
Cancerous cells		
A549	Lung adenocarcinoma	Wild type
A375	Malignant melanoma	Wild type
PANC-1	Pancreatic cancer	Mutant (p.R273H; p.V272A)
HT-29	Colorectal carcinoma	Mutant (p.R273H)
Non-cancerous cells		
HEK293	Embryonic kidney	Wild type

**Table 2:** A comparison of the molecular and in silico ADME profiles of Ezetimibe and nutlin3a.

Properties	Ezetimibe	Nutlin3a
IUPAC name	(3R,4S)-1-(4-fluorophenyl)-3-[(3S)-3-(4-fluorophenyl)-3-hydroxypropyl-4-(4-hydroxyphenyl) azetidin-2-one	4-(4,5-bis(4-chlorophenyl)-2-(2-isopropoxy-4-methoxyphenyl)-4,5-dihydro-1H-imidazole-1-carbonyl) piperazin-2-one
Estimated free energy of binding/docking score (Glide v2021-2)	−7.919 kcal/mol	−6.359 kcal/mol
Molecular weight	409,433 g/mol	581,494 g/mol
Hydrogen bond acceptor	5	5
Hydrogen bond donor	2	1
Rotatable bonds	6	8
Rule of five (No. of violations)	1	4
ClogP	4.330	4.560
Solubility (SILICOS-IT)	2.55 × 10^−5^ mg/mL	4.12 × 10^−7^ mg/mL
Blood–brain barrier (ADMETSAR probability)	0.907	0.740
Human intestinal absorption (ADMETSAR probability)	0.990	1.000
Carcinogens (ADMETSAR probability)	0.833	0.636
Acute oral toxicity (ADMETSAR probability)	0.589	0.661
Aqueous solubility (logS) (ADMETSAR)	−3.876	−3.282
Rat acute toxicity (LD_50_, mol/kg) (ADMETSAR)	2.498	2.591
Solvent accessibility or polar surface area (Å^2^)	60.77 Å^2^	83.47 Å^2^
Binding residues (Mdm2-p53BD)	VAL93, LYS94, GLN72, GLY58, PHE86, ILE103, LEU82, PHE91, LEU57, LEU54, ILE99, TYR100, TYR67, MET62, ILE61	VAL93, GLN72, GLY58, LEU54 ILE99, TYR100, MET62, ILE61

## Data Availability

All data generated or analyzed during this study are included in this published article.

## Supplementary Materials

The following supporting information can be downloaded at: https://www.mdpi.com/article/10.3390/biomedicines13010195/s1. Supplementary Figure S1A: Ezetimibe dependence on Mdm2. Cell viability of A549, A375, HT-29, and HEK293 cells was investigated after treatment with siMDM2 alone (black) and combined with ezetimibe at IC50 concentrations (red). The x-axis represents the concentrations of siMDM2 (0 to 10 nM), and the y-axis shows the percentage of cell viability relative to untreated controls. Cell viability was determined using the MTT assay, and all experiments were performed in triplicate. The graph shows the raw data depicting the trends during the experiment.
