# Supplementary material for: Ezetimibe Anticancer Activity via the p53/Mdm2 Pathway
Source: Biomedicines. 2025 Jan 14;13(1):195. doi: 10.3390/biomedicines13010195 (PMC11761875; doi:10.3390/biomedicines13010195)
Supplement: Supplementary file 1 [file biomedicines-13-00195-s001.zip › biomedicines-3332536-supplementary.pdf]

Supplementary Figure(s)

Supplementary figure S1A

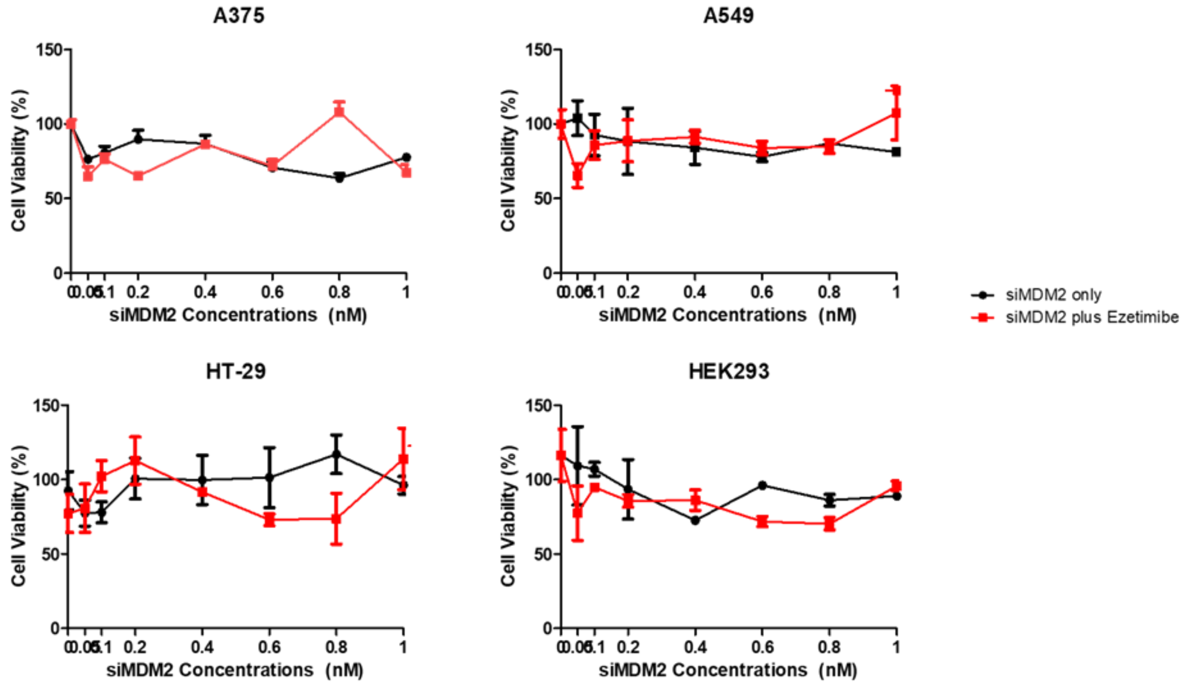

Figure S1A. Ezetimibe dependence on Mdm2. *Cell viability of A549, A375, HT-29, and HEK293 cells was investigated after treatment with siMDM2 alone **(black)** and combined with ezetimibe at IC50 concentrations **(red).** The x-axis represents the concentrations of siMDM2 (0 to 10 nM), and the y-axis shows the percentage of cell viability relative to untreated controls. Cell viability was determined using the MTT assay, and all experiments were performed in triplicate. The graph shows the raw data depicting the trends during the experiment.*
